# Impact of guideline-recommended versus non-guideline-recommended β-blocker and Doppler echocardiographic parameters on 1-year mortality in Thai ischemic cardiomyopathy patients: A prospective multicenter registry

**DOI:** 10.1186/s12872-019-01311-4

**Published:** 2020-01-09

**Authors:** Nattawut Wongpraparut, Sarawut Siwamogsatham, Tomorn Thongsri, Pornchai Ngamjanyaporn, Arintaya Phrommintikul, Kompoj Jirajarus, Tarinee Tangcharoen, Kid Bhumimuang, Pinij Kaewsuwanna, Rungroj Krittayaphong, Rungtiwa Pongakasira, Harvey D. White

**Affiliations:** 1 Division of Cardiology, Department of Medicine, Faculty of Medicine Siriraj Hospital, Mahidol University, 2 Wanglang Road, Bangkoknoi, Bangkok, 10700 Thailand; 2 Department of Medicine, Faculty of Medicine, Chulalongkorn University, Bangkok, Thailand; 3 Department of Medicine, Buddhachinaraj Hospital, Phitsanulok, Thailand; 4 Department of Medicine, Chonburi Hospital, Chonburi, Thailand; 5 Department of Internal Medicine, Faculty of Medicine, Chiang Mai University, Chiang Mai, Thailand; 6 Department of Medicine, Surat Thani Hospital, Surat Thani, Thailand; 7 Department of Internal Medicine, Faculty of Medicine Ramathibodi Hospital, Mahidol University, Bangkok, Thailand; 8 Faculty of Medicine, Thammasat University Hospital, Pathum Thani, Thailand; 9 Department of Internal Medicine, Maharaj Nakhon Ratchasima Hospital, Nakhon Ratchasima, Thailand; 10 Her Majesty’s Cardiac Center, Faculty of Medicine Siriraj Hospital, Mahidol University, Bangkok, Thailand; 11 Green Lane Cardiovascular Service, Auckland City Hospital, Auckland, New Zealand

**Keywords:** Thailand guideline-recommended β-blocker, Doppler echocardiography, 1-year mortality, Ischemic cardiomyopathy

## Abstract

**Background:**

Ischemic cardiomyopathy is a high-cost, resource-intensive public health burden that is associated with a 1-year mortality rate of about 16% in western population. Different in patient ethnicity and pattern of practice may impact the clinical outcome. We aim to determine 1-year mortality and to identify factors that significantly predicts 1-year mortality of Thai patients with ischemic cardiomyopathy.

**Methods:**

This prospective multicenter registry enrolled consecutive Thai patients that were diagnosed with ischemic cardiomyopathy at 9 institutions located across Thailand. Patients with left ventricular function < 40% and one of the following criteria were included: 1) presence of epicardial coronary stenoses > 75% in the left main or proximal left anterior descending artery or coronary angiography, and/or two major epicardial coronary stenoses; 2) prior myocardial infarction; 3) prior revascularization by coronary artery bypass graft or percutaneous coronary intervention; or, 4) magnetic resonance imaging pattern compatible with ischemic cardiomyopathy. Baseline clinical characteristics, coronary and echocardiographic data were recorded. The 1-year clinical outcome was pre-specified.

**Results:**

Four hundred and nineteen patients were enrolled. Thirty-nine patients (9.9%) had died at 1 year, with 27 experiencing cardiovascular death, and 12 experiencing non-cardiovascular death. A comparison between patients who were alive and patients who were dead at 1 year revealed lower baseline left ventricular ejection fraction (LVEF) (26.7 ± 7.6% vs 30.2 ± 7.8%; *p* = 0.021), higher left ventricular end-diastolic volume (LVEDV) (185.8 ± 73.2 ml vs 155.6 ± 64.2 ml; *p* = 0.014), shorter mitral valve deceleration time (142.9 ± 57.5 ml vs 182.4 ± 85.7 ml; *p* = 0.041), and lower use of statins (94.7% vs 99.7%; *p* = 0.029) among deceased patients. Patients receiving guideline-recommended β-blockers had lower mortality than patients receiving non-guideline-recommended β-blockers (8.1% vs 18.2%; *p* = 0.05).

**Conclusions:**

The results of this study revealed a 9.9% 1-year mortality rate among Thai ischemic cardiomyopathy patients. Doppler echocardiographic parameters significantly associated with 1-year mortality were LVEF, LVEDV, mitral E velocity, and mitral valve deceleration time. The use of non-guideline-recommended β-blockers rather than guideline recommended β-blockers were associated with increased with 1-year mortality. Guidelines recommended β-blockers should be preferred.

**Trial registration:**

Thai Clinical Trials Registry TCTR20190722002. Registered 22 July 2019. “Retrospectively registered”.

## Background

The term ischemic cardiomyopathy was introduced by Burch, et al. in 1970 to describe the cause and effect relationship between coronary artery disease (CAD) and severe myocardial dysfunction [1]. Left ventricular (LV) dysfunction from CAD called “ischemic cardiomyopathy” may not only be caused by an acute event such as myocardial infarction (MI) or a consequent from prior MI and scar formation but also may be caused by prolonged ischemia due to chronic CAD and hibernating myocardium [2, 3]. Mortality caused by acute coronary syndrome has decreased significantly over the last 20 years due to advancements in medical, interventional, and surgical treatment. Early reperfusion in ST-elevation myocardial infarction (STEMI) decreases mortality, reduces cardiogenic shock, and preserves LV function [4–9]. However, data from a Thai percutaneous coronary intervention (PCI) registry revealed that a substantial proportion of STEMI patients do not receive timely reperfusion therapy [9]. Patients that fail to receive timely reperfusion therapy, but survive STEMI are likely to develop LV dysfunction due to ischemic cardiomyopathy. Ischemic cardiomyopathy is a high-cost, resource-intensive public health burden that is associated with a 5-year mortality rate of about 40% [10]. Our review of the literature revealed no published data relating to ischemic cardiomyopathy-related mortality in Thailand.

Accordingly, the aim of this study was to establish the first prospective multicenter registry in ischemic cardiomyopathy in Thailand, to determine the 1-year mortality rate, and to identify the factors including type of β blockers used, according to the guidelines [11] that significantly predict 1-year mortality in this patient population.

## Methods

This prospective multicenter registry enrolled consecutive patients diagnosed with ischemic cardiomyopathy at 9 medical centers located across Thailand from December 2014 to November 2015. The protocol for this study was approved by the institutional review boards of all participating centers, and study participants provided written informed consent. This study complied with the principles set forth in the Declaration of Helsinki (1964) and all of its subsequent amendments.

Patients aged greater than 18 years with LV function less than 40% by echocardiogram, magnetic resonance imaging (MRI), LV ventriculogram, or thallium scan within 1 year were included if they satisfied one or more of the following criteria:

1. Presence of epicardial coronary stenoses > 75% in the left main or proximal

left anterior descending artery (LAD) by coronary angiogram, and/or presence of two major epicardial coronary stenoses > 75%

2. Prior history of MI
3. Prior history of revascularization by coronary artery bypass graft (CABG) or percutaneous coronary intervention (PCI)
4. Magnetic resonance imaging (MRI) pattern compatible with ischemic cardiomyopathy

Patients were excluded if they met one of the following criteria:

1. History of MI within 30 days when assessing LV function
2. History of acute coronary syndrome within 30 days when assessing LV function
3. History of significant valvular stenosis or regurgitation that may explain LV dysfunction
4. Currently enrolled in one or more blinded clinical trials
5. Life expectancy less than 1 year
6. Lost to follow-up
7. Refusal to participate
8. Unstable hemodynamic status

Baseline clinical characteristics, angiographic data, procedural characteristics, and periprocedural events were obtained from patients and patient medical records. Patient data was entered into a case record form (CRF) via a web-based system. CRF data was submitted to the Research Unit of the Division of Cardiology, Faculty of Medicine Siriraj Hospital, Mahidol University on the 1st day of every month during the study period. Periodic data verification was performed by primary investigators and nurse coordinators from the Faculty of Medicine Siriraj Hospital. Site monitoring was periodically performed at each site. Patients were followed-up every 6 months for 60 months.

Guideline-recommended β-blockers were defined as Bisoprolol, Carvediolol, Metoprolol succinate, Nebivolol. Non-guideline-recommended β-blockers were defined as Atenolol, Metoprolol tartrate, Propanolol [11].

### Statistical analysis

Patient baseline characteristics are presented using descriptive statistics. Continuous variables are expressed as median (minimum, maximum) or mean ± standard deviation (SD). Categorical variables are expressed as number and percentage. Bivariate analysis of clinical events and baseline, angiographic, and procedural characteristics were performed for categorical variables using the mean of crosstabs, and for continuous variables using comparison of means. Chi-square and Fisher’s exact tests were used to compare each characteristic of interest with clinical events. Those results were expressed as number and percentage (%) for categorical variables, and as mean ± SD for continuous variables. A *p*-value less than 0.05 was considered statistically significant. All statistical analyses were performed using SPSS Statistics version 19.0 (SPSS, Inc., Chicago, IL, USA).

## Results

Four hundred and nineteen patients were enrolled. The mean age of patients was 65.08 ± 11.30 years, and 73% were male. The Thailand Universal Coverage Scheme (UCS) (189 patients, 45.1%) and the Thailand Civil Servant Medical Benefit Scheme (CSMBS) (175 patients, 41.8%) accounted for the majority of reimbursement status. More than half of the patients had a history of prior MI (259 patients, 61.8%). Baseline demographic, clinical, electrocardiographic, and echocardiographic characteristics of patients are shown in Table 1. Two hundred and twenty-six patients (53.9%) had history of PCI. Other previous procedures included CABG (21.5%) and automated implantable cardioverter-defibrillator (AICD) placement (10.3%). The mean ejection fraction (EF) was 29.9 ± 7.7% by Simpson’s method, and 31.6 ± 9.4% by Teicholz’s method. Mean fasting blood sugar was 118.8 ± 37.3 units, mean LDL was 100.2 ± 44.0 mg/L, and the median NT-proBNP was 1470 (38–12,399) units. Of the 410 patients who were taking antiplatelet medication, aspirin was the most common (94.4%), followed by clopidogrel (40.7%). Warfarin was prescribed in 17.3%. Seventy-six percent of patients received beta-blockers, of which carvedilol was the most commonly used (228, 71.7%). 69.2% of patients received either angiotensin converting enzyme-inhibitor (ACE-I) or angiotensin II receptor blockers (ARB).

**Table 1:** Baseline demographic, clinical, electrocardiographic, and echocardiographic characteristics

Characteristics	(*N* = 419)
Demographic data	
Age (years), mean ± SD	65.08 ± 11.30
Male, n (%)	306 (73.0%)
Diabetes, n (%)	181 (43.2%)
Hypertension, n (%)	315 (75.2%)
Chronic kidney disease, n (%)	123 (29.4%)
Dyslipidemia, n (%)	363 (86.6%)
Chronic stable angina, n (%)	34 (8.1%)
History of myocardial infarction, n (%)	259 (61.8%)
Stroke (ischemic), n (%)	38 (9.1%)
Peripheral vascular disease, n (%)	18 (4.3%)
NYHA FC, n (%)	
1	82 (20.6%)
2	238 (59.8%)
3	68 (17.1%)
4	10 (2.5%)
Electrocardiographic data	
Rhythm, n (%)	342 (87.7%)
Sinus Rhythm, n (%)	33 (8.5%)
Atrial fibrillation n (%)	
Rate (bpm), mean ± SD	76.4 ± 18.5
Systolic Blood pressure mmHg	123.5 ± 20.4
Diastolic Blood pressure mmHg	70.6 ± 12.7
Echocardiographic data	
LVEF (%) by Simpson’s method, mean ± SD	29.99 ± 7.68
LVEF (%) by Teicholz’s method, mean ± SD	31.59 ± 9.42
LVESD (mm) by M-mode, mean ± SD	52.90 ± 17.04
LVEDD (mm) by M-mode, median (min, max)	62.00 (0.58, 560.00)
Mitral E wave velocity, mean ± SD m/s	70.68 ± 34.28
Mitral A wave velocity, mean ± SD m/s	65.11 ± 32.11
LV thrombus, n (%)	20 (5.2%)

Abbreviations: *SD* standard deviation; *NYHA FC* New York Heart Association Functional Classification; *bpm* beats per minute; *LVEF* left ventricular ejection fraction; *LVESD* left ventricular end-systolic diameter; *LVEDD* left ventricular end-diastolic diameter

### One-year clinical outcomes

Thirty-nine patients (9.9%) had died at 1-year with 27 (69.2%) experiencing cardiovascular death, and 12 (30.8%) experiencing non-cardiovascular death. Congestive heart failure occurred in 31 patients (7.4%), and non-fatal MI occurred in 6 patients (1.4%). The causes of cardiovascular death were mostly progressive heart failure, arrhythmia, sudden cardiac death, and fatal MI.

### Clinical predictors of 1-year mortality

Patients were classified into two groups at the 1-year time point – dead (group A) or alive (group B). A comparison of baseline characteristics between groups revealed that a significantly higher proportion of patients in group A had a higher New York Heart Association Functional Classification level than the proportion of patients in group B (*p* = 0.012).

The baseline rhythm demonstrated no impact on 1-year mortality; however, mortality was higher among patients with prior inferior myocardial infarction.

Left ventricular ejection fraction (LVEF) was significantly lower in group A than in group B (26.7 ± 7.6% vs. 30.2 ± 7.8%, respectively; *p* = 0.021). Left ventricular end-systolic volume (LVESV) (130.9 ± 56.8 ml vs. 111.6 ± 50.9 ml; *p* = 0.046), left ventricular end-diastolic volume (LVEDV) (185.8 ± 73.2 ml vs. 155.6 ± 64.2 ml; *p* = 0.014), and mitral E velocity (89.7 vs. 64.1 m/s; *p* = 0.040) were all also higher in group A than in group B. Previous history of cardiovascular symptoms, electrocardiographic findings, and echocardiographic findings between patients in group A and patients in group B is shown in Table 2.

**Table 2:** Comparison of Previous history/findings between patients who died and who alive at 1 year

Previous history/findings	Died at 1 year (*n* = 39)	Alive at 1 year (*n* = 357)	*p*-value
Chronic stable angina, n (%)	4 (10.3%)	29 (8.1%)	0.552
Myocardial infarction, n (%)	26 (66.7%)	221 (61.9%)	0.560
Peripheral vascular disease, n (%)	3 (7.7%)	12 (3.4%)	0.175
NYHA FC 1, n (%)	4 (11.1%)	74 (21.7%)	
NYHA FC 2, n (%)	18 (50.0%)	209 (61.3%)	0.012
NYHA FC 3, n (%)	12 (33.3%)	52 (15.2%)	
NYHA FC 4, n (%)	2 (5.6%)	6 (1.8%)	
Baseline HR (bpm), mean ± SD	78.4 ± 17.1	77.1 ± 32.6	0.817
Target HR at 1 year (bpm), mean ± SD	82.3 ± 16.3	73.3 ± 14	0.096
Baseline Systolic Blood Pressure, mmHg	115.2 ± 23.2	124.4 ± 19.8	0.007
Baseline Diastolic Blood Pressure, mmHg	66.9 ± 11.7	71.2 ± 12.7	0.044
Systolic Blood pressure at 1 year, mmHg	111.8 ± 11.2	123.2 ± 19.2	0.094
Diastolic Blood pressure at 1 year, mmHg	67 ± 10.4	70.4 ± 12	0.423
Electrocardiographic findings			
Sinus Rhythm, n (%)	31 (83.8%)	291 (87.7%)	0.546
Atrial fibrillation n (%)	5 (13.5%)	28 (8.4%)	
Rate (bpm), mean ± SD	78.38 ± 15.65	76.03 ± 18.90	0.468
PR interval (ms), median (max, min)	177 (104, 999)	175 (0, 909)	0.320
QRS complex duration (ms), mean ± SD	114.35 ± 30.77	113.64 ± 27.51	0.883
Inferior leads (ІІ or ІІІ or aVF), n (%)	14 (63.6%)	88 (42.1%)	0.053
Anterior leads (І or aVL), n (%)	2 (9.1%)	35 (16.7%)	0.542
Lateral leads (V2-V5), n (%)	14 (63.6%)	150 (71.8%)	0.424
Echocardiographic findings			
LVEF (%) by Simpson’s method, mean ± SD	26.7 ± 7.6	30.2 ± 7.8	0.021
LVESV, mean ± SD ml	130.96 ± 56.83	111.63 ± 50.98	0.046
LVEDV, mean ± SD ml	185.8 ± 73.2	155.6 ± 64.2	0.014
LVEF (%) by Teicholz’s method, mean ± SD	29.27 ± 10.70	32.01 ± 9.25	0.098
Mitral E wave velocity units, median (max, min) m/s	89.7 (11.1, 250)	64.1 (0.32, 167)	0.040
Mitral A wave velocity, median (max, min) m/s	61.6 (5.68, 124.2)	66.1 (0.32, 149)	0.753
Mitral deceleration time, mean ± SD ms	142.9 ± 57.5	182.4 ± 85.7	0.041
RVSP mmHg, mean ± SD	45.58 ± 18.45	40.64 ± 17.42	0.186
LV thrombus, n (%)	3 (8.3%)	15 (4.5%)	0.402

A *p*-value< 0.05 indicates statistical significance

Abbreviations: *NYHA FC* New York Heart Association Functional Classification; *bpm* beats per minute; *SD* standard deviation; *ms* milliseconds; *LBBB* left bundle branch block; *LVEF* left ventricular ejection fraction; *LVESV* left ventricular end-systolic volume; *LVEDV* left ventricular end-diastolic volume; *RVSP* right ventricle systolic pressure

**Fig. 1:**
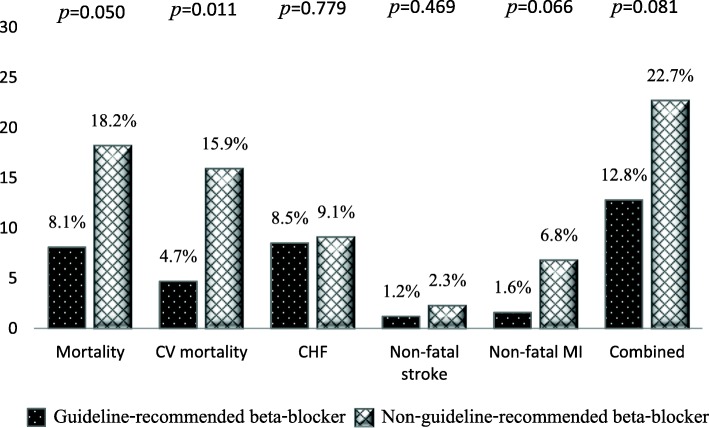
Event rates of all-cause mortality, cardiovascular (CV) mortality, congestive heart failure (CHF), non-fatal stroke, and non-fatal myocardial infarction (MI) in patients who received guideline-recommended β-blockers and patients who received non-guideline-recommended β-blockers. The use of non-guideline-recommended β-blockers rather than guideline recommended β-blockers were associated with increased with 1-year CV mortality (*P* < 0.05)

Medication use compared between patients who died and patients who were alive at 1 year is shown in Table 3. No significant difference was observed between groups for the rate of use of ACE inhibitors, antiplatelets, diuretics, or ivabradine between survivors and those who died at 1 year. Patients in group A had significantly lower statin use (94.7% vs. 99.7%; *p* = 0.029), despite significantly higher use of non-guideline-recommended β-blocker. Patients receiving statin had lower mortality than patients not receiving statin (9.8% vs. 66.7%, respectively; *p* = 0.029) and significantly higher use of non-guideline-recommended β-blockers. Patients receiving guideline-recommended β-blocker had lower mortality than patients receiving non-guideline-recommended β-blocker (8.1% vs. 18.2%, respectively; *p* = 0.050). Event rates who received guideline-recommended β-blockers and patients who received non-guideline-recommended β-blockers are shown in Fig. 1.

**Table 3:** Comparison of baseline medication between patients who died and who were alive at 1 year

Medications	Died at 1 year (*n* = 39) n (%)	Alive at 1 year (*n* = 357) n (%)	*p*-value
Antiplatelet	37 (94.9%)	350 (98.0%)	0.219
Aspirin	34 (91.9%)	332 (94.9%)	0.439
Clopidogrel	13 (35.1%)	143 (40.9%)	0.500
Ticagrelor	2 (5.4%)	7 (2.0%)	0.209
Warfarin	9 (24.3%)	58 (16.6%)	0.236
Beta-blocker	29 (74.4%)	273 (76.5%)	0.769
Bisoprolol	1 (3.4%)	34 (12.5%)	0.223
Metoprolol succinate	0 (0.0%)	6 (2.2%)	1.000
Metoprolol tartrate	5 (17.2%)	24 (8.8%)	0.176
Carvedilol	20 (69.0%)	197 (72.2%)	0.716
Nebivolol	0 (0.0%)	0 (0.0%)	–
Atenolol	1 (3.4%)	10 (3.7%)	1.000
Propranolol	2 (6.9%)	2 (0.7%)	0.047
Non-guideline-recommended	8 (27.6%)	36 (13.2%)	0.050
beta-blocker (metoprolol tartrate or atenolol or propanolol)			
ACE inhibitors	16 (41.0%)	154 (43.1%)	0.800
ARB	11 (28.2%)	99 (27.7%)	0.950
Nitrates	12 (30.8%)	116 (32.5%)	0.827
Hydralazine	3 (7.7%)	34 (9.5%)	1.000
Trimetazidine	3 (7.7%)	26 (7.3%)	1.000
Ivabradine	3 (7.7%)	8 (2.2%)	0.084
Diuretics	30 (76.9%)	255 (71.4%)	0.468
Digitalis	7 (17.9%)	42(11.8%)	0.302
Statins	36 (94.7%)	333 (99.7%)	0.029
Insulin	8 (57.1%)	47 (41.2%)	0.256
Sulfonylureas	4 (28.6%)	57 (50.0%)	0.130
Biguanides	6 (42.9%)	52 (45.6%)	0.845
Thiazolidinediones	0 (0.0%)	3 (2.6%)	1.000

A *p*-value< 0.05 indicates statistical significance

Abbreviations: *ACEI* angiotensin-converting enzyme inhibitors; *ARB* angiotensin receptor blockers

Previous history of cardiovascular intervention shown in Table 4. No significant difference was observed between groups for the rate of use of AICD, cardiac resynchronization therapy (CRT), cardiac resynchronization therapy defibrillator (CRTD), PCI or CABG between survivors and those who died at 1 year.

**Table 4:** Comparison of previous history of cardiovascular intervention between patients who died and who were alive at 1 year

Cardiovascular intervention	Died at 1 year (*n* = 39) n (%)	Alive at 1 year (*n* = 357) n (%)	*p*-value
AICD	4 (10.3%)	38 (10.6%)	1.000
CRT CRTD	0 (0%) 1 (2.6%)	1 (0.3%) 9 (2.5%)	1.000 1.000
AICD/CRT/CRTD	5 (12.8%)	48 (13.4%)	0.913
PCI	16 (41.0%)	197 (55.2%)	0.092
CABG	9 (23.1%)	79 (22.1%)	0.892

A *p*-value< 0.05 indicates statistical significance

Abbreviations: *AICD* automated implantable cardioverter defibrillator; *CRT* cardiac resynchronization therapy; *CRTD* cardiac resynchronization therapy defibrillator; *PCI* percutaneous coronary intervention; *CABG* coronary artery bypass graft

## Discussion

The 1-year mortality rate among Thai ischemic cardiomyopathy patients in this study was 9.9%. We found baseline New York Heart Association (NYHA), presence of prior inferior MI, higher LVEDV, and mitral E velocity with shortened mitral deceleration time by echocardiogram and Doppler to be predictors of 1-year mortality. Mortality was significantly lower among patients who received guideline-recommended β-blockers and/or statin.

The 1-year mortality rate reported in this study is lower than the rates reported from two prior US studies [12, 13]. Mortality was 18% in the Studies of Left Ventricular Dysfunction (SOLVD) Registry and 16% in the Duke Databank for Cardiovascular Disease in 1997. The lower mortality rate in the present study is likely due to the impact of guideline-recommended therapy in the current era, and differences in patient demographics and lifestyle. This study also revealed high rates of use of antiplatelets, statins, ACE-I/ARBs, and β-blockers.

The most important finding from this registry is the difference in mortality rate between patients who received guideline-recommended versus non-guideline-recommended β-blockers. Non-guideline-recommended β-blockers were associated with increased mortality whereas guidelines-recommended β-blockers were associated with decreased mortality. β-blockers provide a well-established mortality benefit in patients with LV dysfunction [14]. β-blockers are currently prescribed in patients with ischemic cardiomyopathy if there is no contraindication. In Thailand, β-blockers are listed as an essential drug, including both guideline-recommended and non-guideline-recommended β-blockers. However, due to budget limits in some hospitals only non-guideline-recommended β-blockers may be provided assuming that the clinical benefits are class effect. The majority of the non-guideline-recommended β-blockers are generic drugs, so there will likely be no pharmaceutical company sponsored studies conducted to investigate the impact of non-guideline-recommended β-blockers on mortality.

The Cardiac Insufficiency Bisoprolol Study (CIBIS) and CIBIS II demonstrated mortality benefit of bisoprolol in patients with heart failure, regardless of etiology. Bisoprolol also showed benefit relative to cardiovascular death and all-cause hospitalization [15, 16]. The mortality benefit of carvedilol was demonstrated in the 2002 Carvedilol Prospective Randomized Cumulative Survival (COPERNICUS) trial [17]. The Metoprolol CR/XL Randomised Intervention Trial in Congestive Heart Failure (MERIT-HF) trial demonstrated the mortality reduction benefit of metoprolol succinate, especially in sudden death and death due to progressive pump failure [18]. In contrast to the benefits of metoprolol succinate on death, metoprolol tartrate demonstrated only quality of life benefit and exercise capacity benefit in the Metoprolol in Dilated Cardiomyopathy (MDC) trial [19]. Uncertainty as to whether there were class effects of β-blockers was increased by the findings in the Carvedilol Or Metoprolol European Trial (COMET) study [20]. Each β-blocker has different sympatholytic effects on β1, β2, β3 and α receptors. Carvediolol has α1, β1 and β2 antagonist activity. They also differ in their lipophilicity, and membrane stabilizing effects. Nebivolol is a beta-1-selective betablocker with vasodilating properties related to nitric oxide modulation that reduce peripheral vascular resistance [21]. Metoprolol succinate has more even beta-blockade over 24 h compare with immediate release Metoprolol tartrate and the target dose can be increased to 200 mg once per day compare with 50 mg three times per day [22]. These differences in pharmacologic properties may lead to different outcomes in heart failure patients.

There is limited evidence that supports the use of non-guideline-recommended β-blockers, such as propranolol or atenolol, for mortality reduction in patients with ischemic cardiomyopathy and reduced EF.

Our comparisons between guideline-recommended and non-guideline-recommended β-blocker found mortality to be significantly lower in patients who received guideline-recommended β-blocker, even though there was no difference between groups for either target heart rate or blood pressure. The results of this study emphasize the importance of selecting β-blockers that are evidence-based and guideline-recommended.

From a healthcare cost standpoint, non-guideline-recommended β-blockers are more affordable, but the cost of overall treatment is likely to be higher, because non-guideline-recommended β-blockers do not significantly reduce cardiovascular events and death. The findings in this study in relation to the type of β-blocker that should be used in this ischemic cardiomyopathy will be of benefit to both clinicians and healthcare policymakers. This study supports the concept that only guideline-recommended β-blockers should be prescribed for patients with ischemic cardiomyopathy. These data also strongly suggests that these drugs should be approved for inclusion on the drug lists of Thailand’s healthcare coverage schemes.

We found NYHA class to be a significant predictor of 1-year mortality, which is similar to the results of two prior studies [23, 24]. Van de Broek SA, et al. found NYHA class and peak oxygen consumption (VO2) to be significantly worse in stable NYHA class II and III heart failure patients with LVEF < 40% who died within a 2-year follow-up period [23]. Coronary artery disease, LVEF, and NYHA III are independent predictors of mortality in the patients with mild to moderate symptomatic heart failure as reported by Scrutinio D et al. [24].

Interestingly, we found a trend for the presence of inferior MI but not anterior MI to predict death by 1 year (*p* = 0.052). This may be explained by a bias caused by our inclusion criteria that defined ischemic cardiomyopathy by presence of epicardial coronary stenoses greater than 75% in the left main or proximal left anterior descending artery (LAD), and/or presence of two major epicardial coronary stenoses > 75%. Therefore, the majority of patients enrolled in our study had coronary stenoses of LAD or infarction in the anterior wall territory by inclusion criteria. The additional presence of inferior infarction may influence mortality due to a larger area of MI involvement.

LVEF was also found to be a significant predictor of 1-year mortality as previously described [13, 25]. Left ventricular end-diastolic volume was also significantly higher in patients with 1-year mortality. Several echocardiographic hemodynamic parameters predicted 1-year mortality. Elevation of mitral E velocity, indicating markedly elevated left ventricular end-diastolic pressure (LVEDP) and high left atrial pressure, was significantly higher in patients who died.

Patients with low ejection fractions have diastolic dysfunction as their reduction in LV function progresses. In contrast to the reverse E/A ratio that survivors had at 1 year, the patients that had died had pseudo normalization of E/A ratio. By just looking at the pattern of E/A ratio may be confusing. The mitral deceleration time helps interpreting hemodynamic parameters. Mitral valve deceleration time is a noninvasive Doppler echocardiographic parameter that evaluates left ventricular filling pressure, and has been shown to correlate well with pulmonary capillary wedge pressure in patients with reduced left ventricular EF [26]. Also the usefulness of the echocardiographic mitral flow velocities curves has been shown for estimating left ventricular filling pressure in patients with left ventricular dysfunction [27]. Mitral deceleration time < 140 ms predicts cardiovascular mortality in acute MI [28, 29]. In the present study, all patients that died by the 1-year follow-up had a mitral deceleration time of less than 150 ms. High mitral E velocity with a short mitral deceleration time demonstrates a restrictive pattern. The patients who died had pseudo normalization of the E/A ratio with a restrictive pattern of mitral deceleration time indicating further progression of diastolic dysfunction. Combining mitral deceleration time with E/A ratio also predicted 1-year mortality. In this study, we identified echocardiographic and hemodynamic parameters that indicate markedly elevated left ventricular pressure, elevated left atrial pressure, elevated mitral E velocity, and shortened mitral deceleration time. All of these parameters were shown to significantly predict 1-year mortality.

## Conclusions

The results of this study revealed a 9.9% mortality rate among Thai patient with ischemic cardiomyopathy. The Doppler echocardiographic parameters that were found to be significantly associated with 1-year mortality were LVEF, LVEDV, mitral E velocity, and mitral valve deceleration time. The use of non-guideline-recommended β-blockers was associated with increased 1-year mortality.

## Data Availability

The datasets used and/or analysed during the current study are de-identified and available from the corresponding author on reasonable request. Identifying/confidential patient data should not be shared.

## Abbreviations

ACEI: Angiotensin converting enzyme-inhibitor
AICD: Automated implantable cardioverter-defibrillator
ARB: Angiotensin II receptor blockers
CABG: Coronary artery bypass graft
CAD: Coronary artery disease
CHF: Congestive heart failure
CIBIS: The cardiac insufficiency Bisoprolol study
COMET: The Carvedilol or Metoprolol European trial
COPERNICUS: Carvedilol prospective randomized cumulative survival
CRF: Case record form
CSMBS: Thailand civil servant medical benefit scheme
LAD: Left anterior descending artery
LV: Left ventricular
LVEDP: Left ventricular end-diastolic pressure
LVEDV: Left ventricular end-diastolic volume
LVEF: Left ventricular ejection fraction
LVESV: Left ventricular end-systolic volume
MDC: The Metoprolol in dilated cardiomyopathy
MERIT-HF: The Metoprolol CR/XL randomised intervention trial in congestive heart failure
MI: Myocardial infarction
MRI: Magnetic resonance imaging
NYHA: New York heart association
PCI: Percutaneous coronary intervention
SD: Standard deviation
SOLVD: The studies of left ventricular dysfunction
STEMI: ST-elevation myocardial infarction
UCS: The Thailand universal coverage scheme
